# Effect of deep marginal elevation with different intermediate materials on the fracture resistance of direct and indirect final composite restorations: an in vitro study

**DOI:** 10.1038/s41598-026-51161-2

**Published:** 2026-05-23

**Authors:** Rofida Ragab, Rasha Saad, Mona Riad

**Affiliations:** 1 Conservative Dentistry Department, Faculty of Dentistry, Minia University, Minia, Egypt; 2 Conservative Dentistry, Faculty of Dentistry, Cairo University, 11 El-Saraya St, Manial, Cairo, 11553 Egypt

**Keywords:** Beautifil Flow Plus, Fracture resistance, Gingival margin elevation, Resin modified glass ionomer, Tetric N flow, Health care, Materials science, Medical research

## Abstract

To compare an injectable hybrid composite for deep marginal elevation with a resin-modified glass ionomer and a flowable composite, and to assess their effects on the fracture resistance of direct restorations and indirect resin composite inlays at different depths. Ninety non-carious maxillary premolar teeth were selected to receive standardized mesio-occluso-distal (MOD) cavity preparations. The specimens were systematically classified into subgroups and classes as follows: Groups (Restorative Material): Specimens were divided into two main groups (*N* = 45) based on the nano-ceramic resin composite used (Spectra ST): direct restorations or indirect inlay restorations. Subgroups (Intermediate Material): Each group was further divided into three equal subgroups (*N* = 15) based on the intermediate material (B) used: resin-modified glass ionomer (B1:RMGI), flowable resin composite (B2), and injectable hybrid composite (B3).Classes (Gingival Box Depth): Finally, each subgroup was subdivided into three classes (*N* = 5) based on the depth of the gingival box (D) relative to the cementoenamel junction (CEJ): at the CEJ (D1), 2 mm above the CEJ (D2), and 2 mm below the CEJ (D3). Fracture resistance was tested using a universal testing machine. Fracture types were assessed under a stereomicroscope. Selected samples underwent scanning electron microscopy analysis. Data were statistically analyzed using Fisher’s exact test, z-tests, Shapiro-Wilk tests, Levene’s tests, and a three-way ANOVA. direct restorations and indirect inlay restorations did not significantly affect fracture resistance when considered as a standalone factor (*p* = 0.686). For the injectable hybrid composite, the indirect composite inlay exhibited higher fracture resistance at and above the CEJ (*p* < 0.001*). The flowable composite showed a significant difference across depths when used as a direct composite restoration (*p* = 0.025*). For RMGI, the indirect composite inlay was significantly superior only below the CEJ (*p* < 0.001*). The box depth below the CEJ generally reduced fracture resistance. While a comparison between direct restorations vs. indirect composite inlay restorations alone does not dictate fracture resistance, material choice and cavity depth are critical. Indirect composite inlay restorations using injectable hybrid composites provide superior strength at/above the Cementum-Enamel Junction (CEJ), while RMGI excels only below it; greater depth reduces overall resistance.

## Introduction

Advances in adhesive restorative materials have promoted the concept of minimally invasive dentistry. This concept is associated with the “biomimetics” philosophy^1,2^, which advocates tissue preservation and restorative procedures that use materials with physical–mechanical properties similar to those of the missing dental structures and that are guided by biomechanical principles.

Restoring teeth with extensive cavities and intrasulcular proximal margins poses a clinical challenge^3^. Deep gingival margins make several operative steps difficult, including finishing preparation margins, rubber dam isolation, obtaining adequate margin impressions, and detecting and removing excess resin cement during luting procedures^4,5^. To address these limitations, Deep Margin Elevation (DME), also known as “proximal box elevation,” has emerged as a conservative alternative to surgical crown lengthening. Deep subgingival margins are sometimes associated with attachment loss, alveolar bone loss, and the opening of interproximal contacts. Moreover, the gingival marginal level after healing is unpredictable^6,7^.

DME, first introduced by Dietschi and Spreafico^8^, involves relocating the subgingival margin coronally using an adhesive intermediate layer. This relocation facilitates easier impression-taking, ensures proper moisture control during the final restoration, and preserves the supporting periodontal tissues^4,9^.

The management of deep interproximal carious lesions has always been a challenge in clinical practice. Interproximal wall loss complicates restorative procedures by creating large cavities, such as compound class II or MOD lesions, which are more difficult to manage^3^. Restoring these cavities with direct composite risks excessive polymerization shrinkage, which may adversely affect marginal adaptation and, in turn, lead to microleakage and recurrent caries^8^.

In cases with deep interproximal cavities, indirect restorations are a more suitable option because they minimize shrinkage-induced stresses on the compromised tooth. This can improve marginal adaptation, overall fracture resistance, and clinical longevity^10,11^.

As a conservative alternative, Deep Margin Elevation (DME) enables the coronal relocation of the subgingival margin using an adhesive intermediate layer. This technique, rooted in the “open sandwich” approach, simplifies moisture control and preserves sound tooth structure. Following this procedure, isolation is more reliable, and bonding of indirect restorations is more predictable. Additionally, decreasing cavity depth improves access for polymerization, allowing better marginal adaptation and decreasing polymerization shrinkage^12^.

Laboratory studies have consistently favored DME, showing that when resin-based materials are used to elevate the margin, the marginal integrity of the final indirect restoration is comparable to, or in some cases exceeds, that of restorations placed on enamel. Furthermore, clinical trials have shown that periodontal tissues remain healthy when well-polished DME materials are used, provided the biological width is maintained^13^.

The success of this technique depends heavily on the biomechanical stability of the elevation material. Highly filled, flowable composites have become an accepted standard for DME; their low viscosity enables excellent adaptation to the gingival floor, while their physical properties provide a stable base that can withstand polymerization stresses. Flowable composites (e.g., Tetric^®^ N-Flow) may also serve as stress absorbers or stress breakers. Stress absorption is strongly influenced by the elastic modulus and other physical properties of restorative materials^13^.

Laboratory studies have validated the use of resin-modified glass ionomer (RMGI) for this purpose, citing its hydrophilic nature and chemical bond to moist dentin as critical to achieving a predictable cervical seal^14^.

Furthermore, advances in materials science have introduced injectable hybrid composites, such as Beautifil Flow Plus, that combine the adaptability of a flowable composite with the mechanical strength of a traditional hybrid. Research indicates that these materials provide a tight marginal seal and high radiopacity, both of which are essential for long-term radiographic monitoring^15^.

When combined with modern indirect restorations (IRs), such as ceramic or composite inlays and overlays, DME relieves polymerization shrinkage stresses and restores anatomic form^16^.

Given that fracture resistance is a primary determinant of restorative longevity, evaluating how different DME materials influence the structural integrity of the final restoration is essential for establishing a reliable clinical protocol^17^.

Therefore, this in vitro study aims to evaluate the effect of proximal box elevation (PBE) at different depths using three intermediate materials (resin-modified glass ionomer (RMGI), flowable composite (Tetric^®^ N-Flow), and injectable hybrid composite (Beautifil Flow Plus)) on the fracture resistance of maxillary premolars restored with direct restorations and indirect composite inlay restorations.

The null hypothesis was that there would be no significant difference in the fracture resistance of premolars among the three intermediate materials (RMGI, Tetric^®^ N-Flow, and Beautifil Flow Plus) used for deep marginal elevation.

## Materials and methods

### Sample size calculation

For this study, the minimum sample size was set at 56 participants, based on a previous investigation^18^ to ensure consistency in the study design. Using G*Power software (version 3.1.9), the sample size was calculated with a significance threshold of 0.05 and a 95% confidence interval. Although the study was designed to achieve at least 80% power, the specific parameters yielded an actual power of 95.93%. To enhance the validity of the results and account for potential attrition or non-response, the final sample size was increased to 90.

### Ethical approval

The ethical protocol approved by the Minia University Research Ethics Committee was followed during tooth collection. The Oral Surgery Department of Minia University’s Faculty of Dentistry provided the teeth. Each participant provided written informed consent before collection, ensuring that all donors were fully informed about the study’s goals, methods, and potential risks.

This commitment to ethical standards underscores the importance of protecting the rights and well-being of participants throughout the research process. Teeth were extracted for orthodontic reasons, typically due to orthodontic or periodontal issues. To minimize stress on the tooth structure, surgeons used forceps and elevators in a controlled, steady manner. The study was approved by the Faculty of Dentistry’s Research Ethics Committee on 2–12-2024 under number 37/475 and was conducted in compliance with the Declaration of Helsinki.

### Specimens’ preparation and grouping

Ninety non-carious maxillary premolars with intact cusps and walls were used in this in vitro investigation. They were obtained from orthodontic patients at a specialized clinic, aged 18 to 40. Microscopic inspection (e.g., 10× magnification) was used to exclude teeth with obvious cracks, cavities, restorations, or hypoplastic abnormalities. Teeth meeting the inclusion criteria were stored at 4 °C in saline containing 0.1% thymol to inhibit bacterial growth for up to 1 month^19^.

### Standardization of teeth

To ensure uniformity, a digital caliper was used to measure the precise dimensions of each tooth and determine the mean mesio-distal width (7 ± 0.5 mm) and bucco-lingual width (8 ± 0.5 mm).

### Grouping of teeth

The specimens (*n* = 45) were divided into two equal groups based on whether a direct or indirect nano-ceramic resin composite inlay restoration (Spectra ST) was performed. Table 1 lists the materials used in this investigation.

After that, each group was divided into three equal subgroups (*n* = 15) based on the intermediate material: resin-modified glass ionomer (B1), flowable composite (B2), and injectable hybrid composite (B3). Each subgroup was then divided into three classes (*n* = 5) based on the depth of the gingival box (D), assessed at the cementoenamel junction (D1), 2 mm above it (D2), and 2 mm below it (D3).

Teeth in acrylic resin were held in place by a hollow, cylindrical metallic mould with a 25 mm diameter. Usually, the roots were vertically inserted 4 mm below the cementoenamel junction (CEJ) into self-curing acrylic resin blocks (Acrostone, Dental Factory, Egypt). A surveyor (Ney Dental Surveyor, Anaheim, CA, USA) was securely fastened to the mould to ensure accurate centering and alignment of each specimen with the tooth’s long axis.

### Tooth preparation

To minimize inter-operator variability, all specimens were processed by a single operator^20^. A high-speed handpiece (PANA MAX, NSK, Japan) was used to prepare teeth to standardized class II MOD cavity dimensions. The occlusal cavity was prepared to half the intercuspal distance or to a depth of 2 mm from the level of the central groove. For this technique, a cylindrical diamond bur (No. 959KRD, 314, 018, Komet, Lemgo, Germany) was used. The cavities were prepared with an isthmus width of approximately 2 mm (1/2 of the intercuspal distance). The axial walls were tapered 6° to 10° for indirect composite inlay restoration and kept parallel or slightly diverging for direct restorations. For this purpose, a tapered diamond bur (No. 6847KRD, 314,016, Komet, Lemgo, Germany) was used^21^. The depth of the proximal box was categorized based on the depth of the gingival box. Thirty premolars were prepared with the gingival floor at the cementoenamel junction, thirty at 2 mm above it, and thirty at 2 mm below it. All point and line angles were rounded. Following cavity preparation, the cavities were irrigated and dried with water. An Auto Matrix^®^ band (Dentsply) was used to wrap each tooth to create the proper shape and prevent overfilling.

### Application of intermediate material

DME materials are typically applied to subgingival cavities to raise the restorative margin to a supragingival level. They serve as a base, not strictly as a cavity liner, to improve rubber dam isolation and impression-taking for indirect restorations. This layer is not always of uniform thickness, as it depends on the cavity depth.

### Resin-modified glass ionomer application

Thirty teeth with various proximal box preparations were cleaned and dried before applying the resin-modified glass ionomer. After conditioning the dentin surfaces on each tooth for 20 s with GC Fuji Plus conditioner (GC Co., Tokyo, Japan), the teeth were rinsed and gently dried. An amalgamator (Ultramat 2, SDI, Bayswater, VIC, Australia) was used to mix the RMGI capsules for ten seconds. Following mixing, the material was placed into the proximal cervical portion of the cavity by loading the capsule into a gun applicator. Gentle pressure was applied to extrude excess material and obtain a flat, smooth surface. An LED curing light unit (Steplight SL-L, serial No. 03894, Tokyo, Japan) with a light intensity of 1200 mW/cm² was used to light-cure the RM-GIC material for 20 s.

### Tetric N-Flow and Beautifil Flow Plus intermediate material applications

Tetric^®^N-Flow and Beautifil Flow Plus intermediate material applications were applied to 60 premolars; 30 premolars received Tetric^®^N-Flow, and the other 30 received Beautifil Flow Plus. A 37% phosphoric acid gel was applied for 15 s to etch the cementum cavity margins on the gingival, buccal, and palatal surfaces of 20 premolars below the CEJ. The treated surfaces were then rinsed with a strong spray of water for five seconds and properly dried with compressed air.

37% phosphoric acid gel was applied for 15 s to etch the enamel margins on the gingival, buccal, and palatal cavity borders of 40 premolars, both at and above the CEJ. The treated surfaces were then rinsed with a strong water spray for five seconds and properly dried with compressed air.

Then, the dental adhesive (Universal Tetric N-Bond) was applied with a microbrush (KaVo Kerr) for 15 s with agitation, followed by air thinning for 5 s as instructed and light curing for 20 s. The materials (Tetric^®^ N-Flow or Beautifil Flow Plus resin composite) were inserted into the proximal cervical portion of the cavity using a tip from the composite syringe to fill the intermediate area of the proximal cavity. They were then light-cured for 20 s according to the manufacturer’s instructions. Subsequently, the intermediate material was finished.

### The restorative procedures

#### For direct restorations

Twenty-five resin composite inlays were placed directly into premolar cavities prepared by wrapping each tooth with an Auto Matrix^®^ band (Dentsply) to create the proper shape. First, a 37% phosphoric acid gel was applied for 15 s to etch the enamel margins of the cavity. After thoroughly cleaning the treated enamel with a strong stream of water for five seconds, it was carefully dried with compressed air. The adhesive (Universal Tetric N-Bond) was immediately applied with a microbrush and left on for 20 s. To achieve thorough coverage of the enamel and dentin with the adhesive, oil-free compressed air was used until a glossy, stable film was observed. The adhesive was light-cured for 20 s. Then, the nano-ceramic composite was placed in the cavity, starting toward the proximal surface. Layer by layer, the nano-ceramic composite was inserted into the cavity, working its way toward the proximal surface until the cavity was filled, maintaining a uniform 1.5 mm distance between the restoration’s central groove and the prepared tooth’s occlusal surface. To achieve the desired final shape of the anatomical features, fissures, and grooves, each layer was light-cured for 40 s using plastic composite tools and burnishers.

Lastly, flexible discs (Sof-Lex; 3 M ESPE, St. Paul, MN, USA) and fine-grit diamond composite finishing burs (Diamond Composite Finishing Kit, Komet, USA) were used for finishing with water cooling. Polishing discs, brushes, and finishing strips coated with diamond paste (Prisma Gloss, Dentsply, USA) were used for polishing.

For indirect composite inlay restorations, twenty-five resin composite inlays were placed indirectly into premolar cavities prepared as follows: an Auto Matrix^®^ band (Dentsply) was wrapped around each tooth to create the correct shape, and a separating medium was applied to all cavity walls to facilitate removal of the indirect restorations. Using plastic composite tools and burnishers, the nano-ceramic composite was deposited into the cavity, layer by layer, toward the proximal surface. A uniform 1.5 mm distance between the central groove of the restoration and the occlusal surface of the prepared tooth was maintained to standardize the occlusal anatomy of the restorations. This made it easier to achieve the desired final shape of the anatomical features, fissures, and grooves.

Indirect composite inlays were fabricated by layering composite resin onto a die model, pre-curing each layer for 20 s, and applying a final long-duration post-cure (5 min) to maximize polymerization and physical properties^22^. The marginal fit of each restoration on the tooth was examined. Restorations with an unacceptable fit, namely those with a visible marginal opening and/or detectable with a probe (probe No. 8, Dentsply LTD, UK), were excluded, and new restorations were fabricated.

### Luting steps for indirect composite inlay restorations

Preparation of the restoration: Aluminum oxide particles were used for sandblasting at 102.5 bar for approximately 10 s, followed by rinsing and drying. A silane coupling agent was then applied to the sandblasted surface for 60 s and air-dried. Finally, a layer of uncured adhesive was applied to the treated restoration surface. Preparation of the tooth surface: After complete cleaning and rinsing, the enamel was etched with 37% phosphoric acid gel for 30 s; the adhesive was then applied and cured for 20 s. Cement material: The same type of intermediate material (resin-modified glass ionomer, flowable composite, or injectable hybrid composite) was applied to the cavity or restoration, and the inlay was seated under firm pressure. Excess cement was removed with an explorer, and final curing was performed from all aspects (occlusal, buccal, and lingual)^22^.

**Fig. 1:**
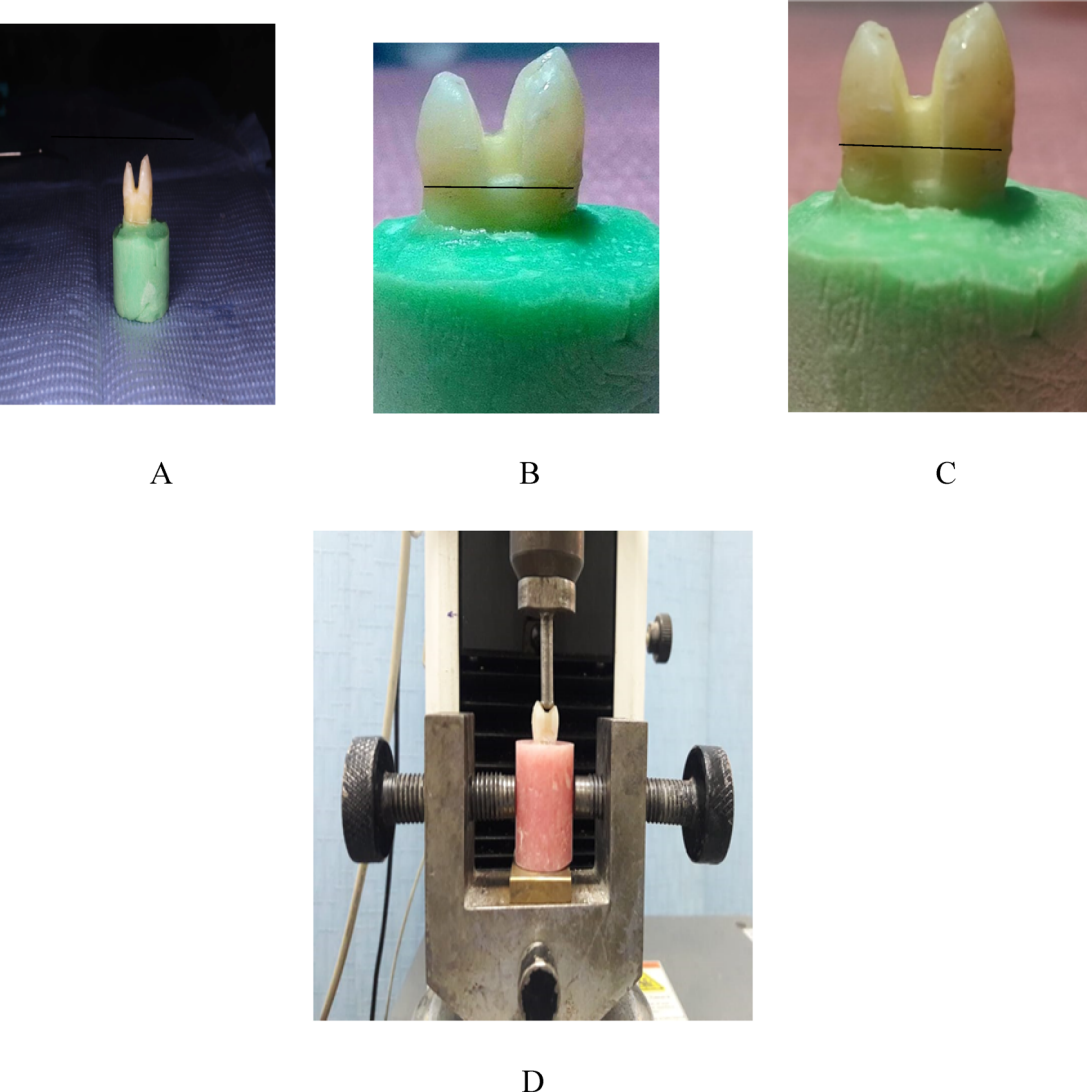
Tooth preparation, restorative placement steps. The drawn line represented CEJ (black solid line). (**A**) cavity preparation with the depth box 2 mm above the CEJ. (**B**) cavity preparation with the box depth at CEJ. (**C**) cavity preparation with the box depth below CEJ. (**D**) fracture resistance testing machine.

All procedures are illustrated in Fig. 1.

**Table 1:** Materials, specifications, chemical composition, manufacturers, and batch numbers.

Material used	Specification	Composition	Manufacturer	Batch number
SPECTRA ST	Universal composite restorative	Methacrylate modified polysiloxane (organically modified ceramic) dimethacrylate resins, ethyl-4 (dimethylamino) benzoate, and bis(4-methyl-phenyl) iodonium hexafluorophosphate. Filler load: 78–80% by weight: Spherical, pre-polymerized SphereTEC fillers (d3,50 ≈ 15 μm), non-agglomerated barium glass, and ytterbium fluoride	Dentsply DeTrey GmbH De-Trey-str. 178,467 Konstanz GERMANY http://www.dentsplysirona.com	221,100,178
Beautifil-Flow PLUS	Fowable-composite	Bis-GMA^1^, TEGDMA^2^, S-PRG ^3^filler based on fluoroboroaluminosilicate, polymerization initiator, pigments, and others Filler Loading (wt%)67.2% – 67.8%,Particle Size (µm) 0.01–4.0	SHOFU INC 11kamitakamatsu-cho, Fukuine, Higashiyama-ku, Kyoto 605–0983, Japan http://www.shofu.com	122,219
GC Fuji II LC capsule	Resin-modified glass ionomer	-powder: fluoro-alumino-silicate glass, initiator, pigment -liquid: methacrylate, water, polyacrylic acid, dimethacrylate, carboxylic acid, initiator, stabilizer Filler Loading (wt%) 40%−50%,Particle Size (µm) 0.4–10	76 − 1 Hasunuma-cho, Itabashi-ku, Tokyo 174–8585, Japan (GC Co., Tokyo, Japan)	240,307 A
Tetric^®^ N-Flow	Fowable- composite	36wt.%dimethacrylate(including TEGDMA), 63wt.%fillers(barium glass, ytterbium trifuorid, highly dispersed silica and mixed oxide) and 1wt.%initiator, stabilizers, and pigment Filler Loading (wt%) 68.2%, Particle Size (µm)0.1–30 (Mean: 5.0)	Ivoclar Vivadent, Schaan, Liechtenstein, USA https://www.ivoclar.com	Z04360
N-Etch	Etchant agent.	Etchant agent.		Z014VZ
Tetric^®^ N-Bond Universal	One-step self-etch adhesive system.	Methacrylates, ethanol, water, highly dispersed silicon dioxide, initiators, and stabilizers.		Z00SNR

1-Bisphenol-A glycidyl ether dimethacrylate, 2-Tri-ethylene glycol dimethacrylate, 3- Surface Pre-Reacted Glass-ionomer.

### Measurement of fracture resistance

The samples were secured in a metal holder and subjected to static loading on a universal testing machine (LARYEE Universal Testing Machine, Jinan, Shandong, China). A 6-mm-diameter custom-made stainless-steel sphere was positioned vertically on the marginal ridge areas, aligned with the long axis of the tooth, to determine fracture strength and failure mode. To prevent excessive load concentrations on the tooth surface, a 0.5-mm-thick aluminum foil layer was placed between the inlay surface and the steel sphere. The load was applied at a crosshead speed of 0.5 mm/min until fracture occurred, and the maximum breaking load for each sample was automatically recorded in Newtons (N) by a computer connected to the testing machine.

### Failure mode analysis

The failure mode for each group was examined using a USB digital microscope (U500x Digital Microscope, Guangdong, China) at a fixed magnification of 25X. Failure mode analysis was classified into three types based on observations with the digital microscope: Type I failure limited to the restoration; Type II failure involving the restoration and the tooth above the CEJ; and Type III failure involving the restoration and the tooth below the CEJ.

### Statistical analysis

Fisher’s exact test was used to analyze the categorical data, followed by pairwise comparisons with multiple z-tests. Categorical results were presented as frequencies and percentages, and numerical data were reported as means and standard deviations. Data distributions were visually examined, and Shapiro-Wilk and Levene’s tests were used to assess normality and homogeneity of variance, respectively. The data were found to be normally distributed and to have homogeneous variances across groups. A three-way ANOVA was used to analyze the fracture-resistance data. The error term from the main ANOVA model was used for simple-effect comparisons. P-values for multiple comparisons were adjusted using the False Discovery Rate (FDR) method. The significance level was set at *p* < 0.05 for all tests. All statistical analyses were performed using R version 4.4.2 for Windows.

## Result

### Fracture resistance

**Table 2:** Effect of different variables and their interactions on fracture resistance (N).

Variable	Sum of squares	Df	Mean square	f-value	*p*-value
Inlay material	937.02	1	937.02	**0.17**	**0.686ns**
Intermediate material	44780.95	2	22390.47	**3.95**	0.024*
Box depth	90306.44	2	45153.22	**7.97**	< 0.001*
Inlay material * Intermediate material	211781.84	2	105890.92	**18.68**	< 0.001*
Inlay material * Box depth	157150.46	2	78575.23	**13.86**	< 0.001*
Intermediate material * Box depth	102615.13	4	25653.78	**4.53**	0.003*
Inlay material* Intermediate material* Box depth	411013.00	4	102753.25	**18.13**	< 0.001*

There was a significant interaction among the three variables tested for fracture resistance (*p* < 0.001). The fracture resistance (N) was significantly affected by the intermediate material (*p* = 0.024), the box depth (*p* < 0.001), and all interaction levels (*p* < 0.01). However, the inlay material, as a standalone factor, did not significantly influence the results (*p* = 0.686).

**Table 3:** Comparisons and summary statistics of fracture resistance (N) for different inlay techniques.

Box depth	Intermediate material	Inlay technique		*p*-value
		Fracture resistance (*N*) (Mean ± SD)		
		Direct composite	Indirect composite	
At CEJ	RMGI	555.14 ± 78.68	535.78 ± 56.10	0.686ns
	Tetric N	546.14 ± 64.48	605.40 ± 94.75	0.217ns
	Beautifil	435.00 ± 108.73	804.85 ± 69.84	< 0.001*
Above CEJ	RMGI	536.12 ± 53.29	490.72 ± 57.71	0.344ns
	Tetric N	510.66 ± 78.04	401.85 ± 29.29	0.025*
	Beautifil	497.98 ± 41.09	951.33 ± 111.84	< 0.001*
Below CEJ	RMGI	381.86 ± 27.40	655.72 ± 124.85	< 0.001*
	Tetric N	591.12 ± 63.52	633.02 ± 33.44	0.382ns
	Beautifil	377.95 ± 73.96	436.50 ± 92.60	0.223ns

*Significant, ns not significant.

Beautifil Flow Plus showed highly significant differences (*p* < 0.001) between the direct and indirect methods at the CEJ and above. Tetric^®^ N-Flow showed a significant difference (*p* = 0.025) above the CEJ, with the direct method outperforming the indirect method. The resin-modified glass ionomer (RMGI) showed a highly significant difference (*p* < 0.001) below the CEJ, favoring the indirect method. At other depths, the differences were not statistically significant (*p* > 0.05).

**Fig. 2:**
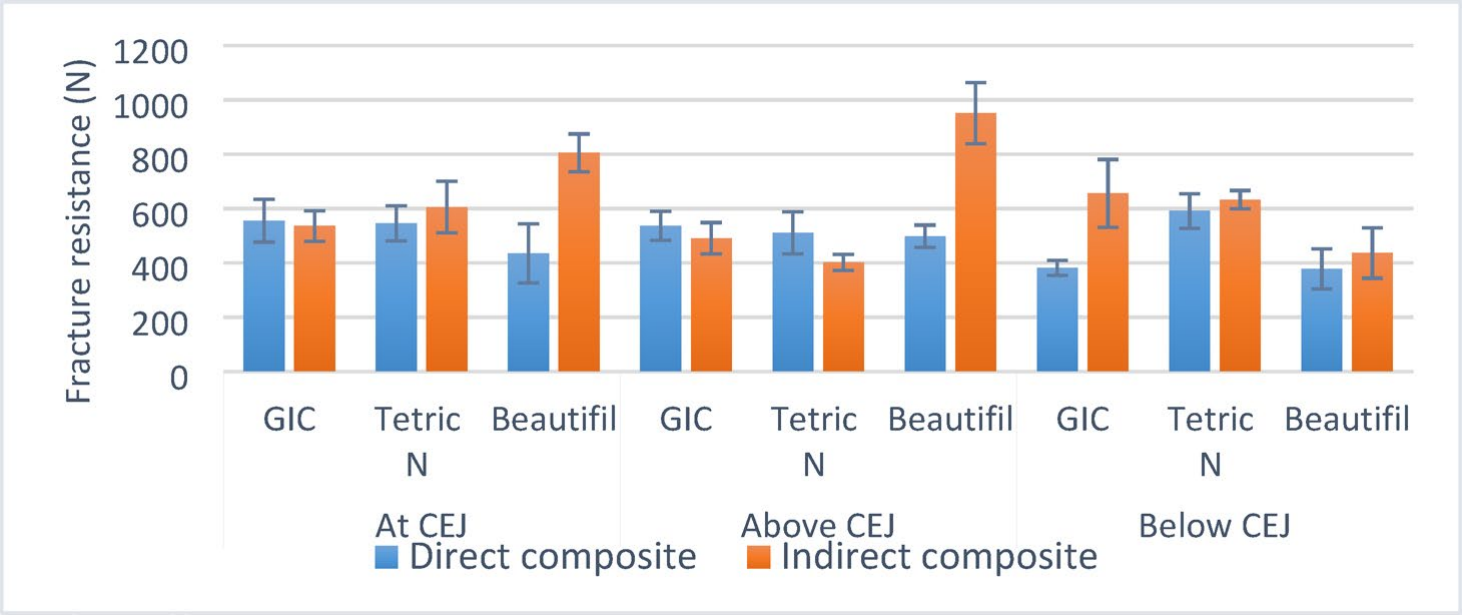
Bar chart showing mean and standard deviation (error bars) values for fracture resistance (N) for different inlay techniques.

Indirect vs. Direct: For Beautifil Flow Plus, the indirect method consistently produced higher fracture resistance at and above the CEJ. Tetric^®^ N-Flow showed the most consistent results across depths when used as a direct composite. For RMGI, the indirect method was significantly superior only below the CEJ (Fig. 2).

The highest fracture resistance was observed with the Beautifil Flow Plus intermediate material when used with an indirect inlay above the CEJ. The lowest fracture resistance was observed with the Beautifil Flow Plus intermediate material when used with a direct restoration below the CEJ.

Reduced box depth below the CEJ generally decreased fracture resistance, as observed with the direct RMGI and Beautifil Flow plus intermediate materials.

- Below the CEJ, RMGI, and Beautifil Flow Plus with direct composites had significantly lower fracture resistance than at other depths.
- For Tetric^®^ N-Flow with direct composites, fracture resistance was significantly lower above the CEJ.
- Below the CEJ, RMGI and Tetric^®^ N-Flow with indirect composite inlays had significantly higher fracture resistance than at other depths.
- All direct composites had type III failures, while the majority of indirect composite inlays had type II failures for RMGI and Tetric^®^ N-Flow at different depths. The differences were not statistically significant.
- For Beautifil Flow, all direct composite samples had type II failures at and above the CEJ and had type III failures below the CEJ for both direct and indirect composite inlays.

**Table 4:** Comparisons and summary statistics of failure modes for different intermediate materials:

Box depth	Inlay material	Failure mode	n(%)			p-value
			GIC	TetricN	Beautifil	
At CEJ	Direct composite	Type(I)	0(0.00%)^A^	0(0.00%)^A^	0(0.00%)^A^	< 0.001*
		Type(II)	0(0.00%)^A^	0(0.00%)^A^	5(100.00%)^B^	
		Type(III)	5(100.00%)^A^	5(100.00%)^A^	0(0.00%)^B^	
	Indirect composite	Type(I)	2(40.00%)	0(0.00%)	0(0.00%)	0.314ns
		Type(II)	0(0.00%)	3(60.00%)	2(40.00%)	
		Type(III)	3(60.00%)	2(40.00%)	3(60.00%)	
Above CEJ	Direct composite	Type(I)	0(0.00%)	0(0.00%)	0(0.00%)	0.165ns
		Type(II)	0(0.00%)	0(0.00%)	3(60.00%)	
		Type(III)	5(100.00%)	5(100.00%)	2(40.00%)	
	Indirect composite	Type(I)	0(0.00%)	0(0.00%)	0(0.00%)	1ns
		Type(II)	3(60.00%)	2(40.00%)	2(40.00%)	
		Type(III)	2(40.00%)	3(60.00%)	3(60.00%)	
Below CEJ	Direct composite	Type(I)	0(0.00%)	0(0.00%)	0(0.00%)	NA
		Type(II)	0(0.00%)	0(0.00%)	0(0.00%)	
		Type(III)	5(100.00%)	5(100.00%)	5(100.00%)	
	Indirect Composite	Type(I)	0(0.00%)	0(0.00%)	0(0.00%)	0.314ns
		Type(II)	2(40.00%)	3(60.00%)	0(0.00%)	
		Type(III)	3(60.00%)	2(40.00%)	5(100.00%)	

NA Not Applicable, Values with different superscripts within the same horizontal row are significantly different, * significant (p < 0.05), ns not significant.

## Discussion

Recent advances in clinical techniques and dental materials have enabled the restoration of deep subgingival cavities in posterior teeth^23^. Deep margin elevation (DME) uses various restorative materials to reposition subgingival margins to a supragingival level, thereby enhancing marginal integrity and bond strength^24^. Periodontal ligament simulation was omitted to simplify the study design, as the primary goal was to compare restorative materials rather than predict clinical longevity. A study by Marchionatti et al. found that the materials used to simulate the periodontal ligament did not affect fracture or bond strength. Therefore, periodontal ligament simulation using the tested materials could be considered optional under the study’s conditions^25^. Using specimens with varying proximal box depths in DME studies simulates diverse, realistic clinical scenarios involving subgingival caries or fractures, thereby testing the versatility and reliability of materials across varying levels of tooth destruction^26,27^.

In the present study, the null hypothesis was rejected; the injectable composite (Beautifil Flow Plus) showed significantly higher fracture resistance than other intermediate materials in indirect restorations when the gingival margin was at or above the CEJ (Table 3). Significant differences were also observed among other intermediate materials used for direct restorations (Table 2).

The superior performance of Beautifil Flow Plus in specific experimental groups (Table 3) may be attributed to its unique filler technology. This finding aligns with Yu et al.^28^, who suggested that the increased stiffness of injectable hybrid composites effectively mitigates tooth-bending damage under occlusal loading. This theory is further supported by our failure mode analysis, which revealed that most fractures in these groups occurred distal to the tooth, with minimal enamel chipping, suggesting a more favorable stress distribution. Furthermore, the specialized resin composition and filler architecture of injectable hybrids provide superior flowability compared to traditional flowable resins, as noted by Basheer et al.^29^. In contrast, the variable performance of Tetric^®^ N-Flow across depths might be explained by its lower elastic modulus, which, according to Yamanel et al.^20,30^, can transmit higher stresses to the underlying tooth structure (Table 4).

The mechanical behavior observed in this study appears fundamentally linked to filler loading. While higher filler loading generally correlates with improved hardness and fracture resistance, as reported by Kim et al. and Garoshi et al.^31,32^, the specific filler type also plays a critical role. For instance, although barium glass fillers are common, Say et al.^33^ suggested they might occasionally reduce surface hardness. This underscores a vital clinical consideration: the choice of a flowable intermediate material must be carefully balanced to prevent the formation of a “weak link” within the restoration complex, which could jeopardize overall structural durability.

An interesting finding was that RMGI outperformed other materials in fracture resistance for direct restorations at the CEJ and indirect restorations below the CEJ (Table 3). This supports the clinical recommendations of Grubbs et al.^34^ and Vertolli et al.^35^ regarding the efficacy of RMGI in DME procedures. The stability of RMGI is likely due to its chemical bonding and elastic modulus, which are similar to those of dentin. However, while RMGI offers biomechanical benefits, its clinical application requires caution due to higher solubility and lower polishability compared to resin composites, as explained by Kielbassa and Philipp^36^.

Regarding the impact of box depth, our findings indicate that extending the margin below the CEJ generally reduces fracture resistance, particularly for RMGI and Beautifil Flow Plus (Table 3). This trend aligns with Vertolli et al.^35^, who reported significantly higher failure rates at cementum borders (90%) than at enamel (10%). Despite these findings, the literature remains divided. While De Assis et al.^37^ argued that DME enhances fracture resistance through improved structural support, other studies by Ilgenstein et al.^38^ and Zhang et al.^23^ found no significant effect of either the DME procedure or the material type. These discrepancies likely stem from variations in proximal extension lengths, loading protocols, and the seating accuracy of the restorations, as explained by Sandoval et al.^39^.

Our findings showed that the majority of fractures were type III (catastrophic), with vertical fractures originating from the occlusal fissure, splitting the restoration, and occurring within the restoration or material for DME. These findings align with Salah and Seilib^40^, who explained that the DME material layer, along with the inlay, may function as a cohesive unit. Therefore, stress is distributed, and DME mainly displays restorable failure modes. These findings also align with Wafaie et al.^41^ and Bresser et al.^42^. These irreparable fractures often result from stress concentration at the delicate cervical borders, where the stress-strain properties of dentin and composite differ significantly.

Conversely, the fiber-reinforced structure of some hybrid composites may hinder crack propagation, as Roshdy et al.^43^ explain. They suggest that material composition is as important as placement depth in determining the restoration’s long-term success (Table 4).

## Limitations

As an in vitro study, it cannot fully replicate the complex dynamics of the oral environment, including pH fluctuations and thermal cycling, and lacks long-term testing and assessment of how different adhesive systems affect the durability of the DME complex.

## Conclusion

While a comparison between direct restorations and indirect composite inlay restorations alone does not dictate fracture resistance, material choice and cavity depth are critical. Indirect composite inlay restorations using injectable hybrid composites provide superior strength at/above the Cementum-Enamel Junction (CEJ), while RMGI excels only below it; greater depth reduces overall resistance.

### Clinical relevance

Deep marginal elevation is a technique that preserves tooth structure rather than performing surgical crown lengthening to increase the crown’s length. It enhances fracture restorability without reducing fracture resistance.

## Data Availability

The datasets used and/or analyzed during the current study are available from the corresponding author on reasonable request.

## Abbreviations

DME: Deep marginal elevation
CEJ: Cemento enamel junction
RMGI: Resin-modified glass ionomer
MOD: Mesio-occluso-distal

## References

[1] Magne, P. & Douglas, W. H. Rationalization of esthetic restorative dentistry based on biomimetics. *J. Esthet. Restor. Dent.* **11**, 5–15. 10.1111/j.1708-8240.1999.tb00371.x (1999). 10.1111/j.1708-8240.1999.tb00371.x 10337285

[2] Grassi, E. D. A. Efeito da elevação da margem gengival e do material restaurador no comportamento em fadiga e distribuição de tensão de molares restaurados com inlays mod. Universidade Estadual Paulista (Unesp). https://repositorio.unesp.br/entities/publication/3ba0c98c-05a9-421c-a169-3f16c6db76a4. (Accessed 13 Mar 2026) (2021).

[3] Veneziani, M. Adhesive restorations in the posterior area with subgingival cervical margins: New classification and differentiated treatment approach. *Eur. J. Esthet. Dent.* **5**, 50–76 (2010). 20305873

[4] Magne, P. & Spreafico, R. C. Deep margin elevation: A paradigm shift. *Am. J. Esthet. Dent.* **2**, 86–96 (2012).

[5] Müller, V. et al. Influence of proximal box elevation technique on marginal integrity of adhesively luted CEREC inlays. *Clin. Oral Investig.* **21**, 607–12. 10.1007/s00784-016-1927-8 (2017). 27507168 10.1007/s00784-016-1927-8

[6] Bertoldi, C. et al. Clinical and histological reaction of periodontal tissues to subgingival resin composite restorations. *Clin. Oral Investig.* **24**, 1001–11. 10.1007/s00784-019-02998-7 (2020). 31286261 10.1007/s00784-019-02998-7

[7] Sarfati, A. & Tirlet, G. Deep margin elevation versus crown lengthening: Biologic width revisited. *Int. J. Esthet. Dent.* **13**, 334–356 (2018). 30073217

[8] Dietschi, D. & Spreafico, R. Current clinical concepts for adhesive cementation of tooth-colored posterior restorations. *Pract. Periodontics Aesthet. Dent.* **10**, 47–54 (1998). 9582662

[9] Da Silva Gonçalves, D., Cura, M., Ceballos, L. & Fuentes, M. V. Influence of proximal box elevation on bond strength of composite inlays. *Clin. Oral Investig.* **21**, 247–54. 10.1007/s00784-016-1782-7 (2017). 26969499 10.1007/s00784-016-1782-7

[10] Nejad, R. M. et al. Fracture behavior of restored teeth and cavity shape optimization: Numerical and experimental investigation. *J. Mech. Behav. Biomedical Mater. Elsevier*. **124**, 104829 (2021). 10.1016/j.jmbbm.2021.104829 34530299

[11] Opdam, N. J. M., Frankenberger, R. & Magne, P. From ‘direct versus indirect’toward an integrated restorative concept in the posterior dentition. *Oper. Dent.* **41**, S27-34 (2016). 26918928 10.2341/15-126-LIT

[12] Zafar, M. S. et al. Biomimetic aspects of restorative dentistry biomaterials. *Biomimetics* **5**, 34 (2020). 32679703 10.3390/biomimetics5030034 PMC7557867

[13] Frankenberger, R. et al. Effect of proximal box elevation with resin composite on marginal quality of ceramic inlays in vitro. *Clin. Oral Investig.* **17**, 177–83. 10.1007/s00784-012-0677-5 (2013). 22358378 10.1007/s00784-012-0677-5

[14] Loguercio, A. D. et al. Jan. Microleakage in class II composite resin restorations: total bonding and open sandwich technique. *J. Adhes. Dentist.* (2002). 12236642

[15] Benetti, A. R. et al. Adhesion and marginal adaptation of a claimed bioactive, restorative material. *Biomater. Investig. Dent.* **6**, 90–8. 10.1080/26415275.2019.1696202 (2019). 31998876 10.1080/26415275.2019.1696202 PMC6964780

[16] Al-Rawi, B. T. Survival of Cast Gold and Ceramic Onlays Placed in a School of Dentistry [Internet] [Master’s Thesis]. https://search.proquest.com/openview/df2e44f9f51bac88c0fd3f11251b3e1f/1?pq-origsite=gscholar&cbl=18750&diss=y. (Accessed 28 Dec 2025) (The University of North Carolina at Chapel Hill, 2020).

[17] Amesti-Garaizabal, A. et al. Fracture resistance of partial indirect restorations made with CAD/CAM technology. A systematic review and meta-analysis. *J. Clin. Med.* **8**, 1932 (2019). 31717610 10.3390/jcm8111932 PMC6912690

[18] Robaian, A. et al. Different designs of deep marginal elevation and its influence on fracture resistance of teeth with monolith zirconia full-contour crowns. *Medicina* **59**, 661 (2023). 37109619 10.3390/medicina59040661 PMC10144512

[19] El-husseiny, S. E., Nagy, R. A. & Haridy, M. F. Effect of resin restorative material type and layering techniques on the fracture resistance of maxillary premolars. *Cuest. Fisioter.* **54**, 2118–29 (2025).

[20] Ata, M. S. M. Fracture resistance of premolars teeth restored by silorane, nanohybrid and two types of fiber-reinforced composite: An: In-vitro: Study. *Tanta Dent. J.* **14**, 216–219 (2017).

[21] de Azevedo Cubas, G. B., Habekost, L., Camacho, G. B. & Pereira-Cenci, T. Fracture resistance of premolars restored with inlay and onlay ceramic restorations and luted with two different agents. *J. Prosthodontic Res.* **55**, 53–59 (2011). 10.1016/j.jpor.2010.07.001 20934401

[22] Torabzadeh, H., Ghasemi, A., Dabestani, A. & Razmavar, S. Fracture resistance of teeth restored with direct and indirect composite restorations. *J. Dentist.* **10**, 417 (2013). PMC4025423 24910649

[23] Zhang, H. et al. Effect of proximal box elevation on fracture resistance and microleakage of premolars restored with ceramic endocrowns. *PLoS. One* **16**, e0252269 (2021). 34038489 10.1371/journal.pone.0252269 PMC8153463

[24] Juloski, J., Köken, S. & Ferrari, M. Cervical margin relocation in indirect adhesive restorations: A literature review. *J. Prosthodontic Res. Jpn. Prosthodontic Soc.* **62**, 273–280 (2018). 10.1016/j.jpor.2017.09.005 29153552

[25] Marchionatti, A. M. E. et al. Influence of periodontal ligament simulation on bond strength and fracture resistance of roots restored with fiber posts. *J. Appl. Oral Sci. SciELO Brasil*. **22**, 450–458 (2014). 10.1590/1678-775720140067 PMC4245758 25466478

[26] Jakab, A. et al. Mechanical performance of extensive restorations made with short fiber-reinforced composites without coverage: a systematic review of in vitro studies. *Polym. MDPI*. **16**, 590 (2024). 10.3390/polym16050590 PMC10934356 38475274

[27] Theisen, C. E. R. et al. Quality of CAD-CAM inlays placed on aged resin-based composite restorations used as deep margin elevation: A laboratory study. *Clin. Oral Investig.* **27**, 2691–703. 10.1007/s00784-022-04841-y (2023). 36622446 10.1007/s00784-022-04841-y PMC10264514

[28] Yu, P. et al. On the wear behavior and damage mechanism of bonded interface: ceramic vs resin composite inlays. *J. Mech. Behav. Biomedical Mater. Elsevier*. **101**, 103430 (2020). 10.1016/j.jmbbm.2019.103430 31557660

[29] Basheer, R. R., Hasanain, F. A. & Abuelenain, D. A. Evaluating flexure properties, hardness, roughness and microleakage of high-strength injectable dental composite: An in vitro study. *BMC Oral Health* **24**, 546. 10.1186/s12903-024-04333-3 (2024). 38730400 10.1186/s12903-024-04333-3 PMC11088093

[30] Yamanel, K., Çaglar, A., Gülsahi, K. & Özden, U. A. Effects of different ceramic and composite materials on stress distribution in inlay and onlay cavities: 3-D finite element analysis. *Dent. Mater. J.* **28**, 661–70 (2009). 20019416 10.4012/dmj.28.661

[31] Kim, K.-H., Ong, J. L. & Okuno, O. The effect of filler loading and morphology on the mechanical properties of contemporary composites. *J. Prosthet. Dent.* **87**, 642–649 (2002). 12131887 10.1067/mpr.2002.125179

[32] Garoushi, S., Vallittu, P. K. & Lassila, L. V. Short glass fiber reinforced restorative composite resin with semi-inter penetrating polymer network matrix. *Dent. Mater.* **23**, 1356–62 (2007). 17204319 10.1016/j.dental.2006.11.017

[33] Say, E. C., Civelek, A., Nobecourt, A., Ersoy, M. & Guleryuz, C. Wear and microhardness of different resin composite materials. *Oper. Dent.* **28**, 628–34 (2003). 14531611

[34] Grubbs, T. D., Vargas, M., Kolker, J. & Teixeira, E. C. Efficacy of direct restorative materials in proximal box elevation on the margin quality and fracture resistance of molars restored with CAD/CAM onlays. *Oper. Dent.* **45**, 52–61 (2020). 31084532 10.2341/18-098-L

[35] Vertolli, T. J. et al. Effect of deep margin elevation on CAD/CAM-fabricated ceramic inlays. *Oper. Dent.* **45**, 608–17 (2020). 32243253 10.2341/18-315-L

[36] Kielbassa, A. M. & Philipp, F. Restoring proximal cavities of molars using the proximal box elevation technique: Systematic review and report of a case. *Quintessence Int.* (2015). 10.3290/j.qi.a34459 26159213

[37] de Assis, F. S. et al. Evaluation of bond strength, marginal integrity, and fracture strength of bulk-vs incrementally-filled restorations. *J. Adhes. Dent.* **18**, 317–323 (2016). 27419244 10.3290/j.jad.a36516

[38] Ilgenstein, I. et al. Influence of proximal box elevation on the marginal quality and fracture behavior of root-filled molars restored with CAD/CAM ceramic or composite onlays. *Clin. Oral. Investig.* **19**, 1021–1028. 10.1007/s00784-014-1325-z (2015). 25248949 10.1007/s00784-014-1325-z

[39] Sandoval, M. J., Rocca, G. T., Krejci, I., Mandikos, M. & Dietschi, D. In vitro evaluation of marginal and internal adaptation of class II CAD/CAM ceramic restorations with different resinous bases and interface treatments. *Clin. Oral. Investig.* **19**, 2167–77. 10.1007/s00784-015-1449-9 (2015). 25877233 10.1007/s00784-015-1449-9

[40] Salah, Z. & Sleibi, A. Effect of deep margin elevation on fracture resistance of premolars restored with ceramic onlay: In vitro comparative study. *J. Clin. Exp. Dent.* **15**, e446 (2023). 37388433 10.4317/jced.60384 PMC10306389

[41] Wafaie, R. A., Ibrahim Ali, A. & Mahmoud, S. H. Fracture resistance of prepared premolars restored with bonded new lab composite and all-ceramic inlay/onlay restorations: Laboratory study. *J. Esthet Restor. Dent.* **30**, 229–239. 10.1111/jerd.12364 (2018). 29368375 10.1111/jerd.12364

[42] Bresser, R. A. et al. Influence of deep margin elevation and preparation design on the fracture strength of indirectly restored molars. *J. Mech. Behav. Biomedical Mater.* **110**, 103950 (2020). 10.1016/j.jmbbm.2020.103950 32957242

[43] Roshdy, B. N., Eltoukhy, R. I., Ali, A. I. & Mahmoud, S. H. Effect of cervical margin relocation with different injectable restorative materials on fracture resistance of molars received MOD CAD / CAM onlay restorations. *J. Esthet Restor. Dent.* **37**, 1522–1529. 10.1111/jerd.13414 (2025). 39825633 10.1111/jerd.13414

